# Hydroxymethylglutaryl Coenzyme a Reductase Inhibitors Differentially Modulate Plasma Fatty Acids in Rats With Diet-Induced-Hyperhomocysteinemia: Is ω-3 Fatty Acids Supplementation Necessary?

**DOI:** 10.3389/fphys.2019.00892

**Published:** 2019-07-16

**Authors:** Tamara Nikolic Turnic, Aleksandra Arsic, Vesna Vucic, Snjezana Petrovic, Danijela Ristic-Medic, Vladimir Zivkovic, Ivan Srejovic, Jovana Jeremic, Tanja Radonjic, Isidora Milosavljevic, Sergey Bolevich, Stefany Bolevich, Dragan Djuric, Vladimir Jakovljevic

**Affiliations:** ^1^Department of Pharmacy, Faculty of Medical Sciences, University of Kragujevac, Kragujevac, Serbia; ^2^Centre of Research Excellence in Nutrition and Metabolism, Institute for Medical Research, University of Belgrade, Belgrade, Serbia; ^3^Department of Physiology, Faculty of Medical Sciences, University of Kragujevac, Kragujevac, Serbia; ^4^Health Center “Dr Milutin Ivkovic”, Belgrade, Serbia; ^5^Department of Human Pathology, 1st Moscow State Medical, University IM Sechenov, Moscow, Russia; ^6^Department of Pathophysiology, 1st Moscow State Medical, University IM Sechenov, Moscow, Russia; ^7^Faculty of Medicine, Institute of Medical Physiology “Richard Burian,” University of Belgrade, Belgrade, Serbia

**Keywords:** high-methione diet, hyperhomocysteinemia, plasma, fatty acid, rats

## Introduction

Altered fatty acid (FA) metabolism is a hallmark of many chronic non-communicable diseases, including atherosclerosis, cardiovascular disease (CVD), diabetes and inflammatory diseases (Lecerf, 2009; Ristic-Medic et al., 2009; Ristić-Medić et al., 2013). Of particular importance is the role of FAs in the formation of eicosanoids, which are a group of signaling molecules involved in immune response (Vučić, 2013). In contrast to the negative effects of saturated (SFA) and *trans* fatty acids, the intake of both monounsaturated (MUFAs) and ω-3 and ω-6 polyunsaturated fatty acids (PUFAs) is associated with beneficial effects on atherosclerosis (Herold and Kinsella, 1986; Flachs et al., 2009). Among other functions, these FAs increase the uptake of circulating LDL-cholesterol by the liver and reduce leukocyte activation, platelet reactivity, lymphocyte proliferation, and blood pressure (Houston, 2007).

Homocysteine (Hcy) is a sulfur amino acid involved in many biological processes (Kapoor and Huang, 2006; Jacobson, 2007). It is firmed during demethylation process of methionine from diet from animal proteins. We know that the Hcy could be present in various forms: free as thiol, in form of sulfide and in form of dimer with other thiols (Metcalf et al., 2003; Faeh et al., 2006). Hcy is a key determinant of the methylation cycle, and hyperhomocysteinemia (Hhcy) is defined as a medical disorder characterized by abnormally high levels (above 15 μmol/L) of circulated homocysteine in the blood. Hhcy could be a marker of endothelial dysfunction and vascular disease as well as disturbance of homeostasis (Hankey and Eikelboom, 1999).

Hcy has a role in atherosclerotic processes, and there are different therapeutic options indicating the slowing progression of these processes in vessels (Loscalzo and Handy, 2014). The most important therapeutic agents in dyslipidaemia are statins or hydroxy-methyglutaryl coenzyme A (HMG-CoA) reductase inhibitors, which are widely used in lowering the cholesterol levels in preventing and treatment of cardiovascular disorders (Harvey and Ferrier, 2011). Also, several dietary components, such as PUFAs, could decrease cardiovascular risk in patients, but there is little data about the relationship between fatty acid status and homocysteine levels.

Previous studies have found that dietary FAs interact with methylenetetrahydrofolate reductase (MTHFR) and enzyme such as methionine adenosyltransferase I, which are alpha (MAT1A) genetic variants for determining plasma Hcy concentrations (Maron et al., 2000; Palinski, 2001). In contrast, acetyl coenzyme A carboxylase and fatty acid synthase, could be involved in regulation of FA biosynthesis, as enzymes and can be simultaneously controlled by HMG-CoA reductase enzyme at the molecular level (Greenwood et al., 2006; Wang et al., 2008). These mentioned mechanisms are associated with Hhcy and statin treatment (Maron et al., 2000; Palinski, 2001; Greenwood et al., 2006; Wang et al., 2008).

The present study examines the levels Hcy and FAs in total plasma lipids from animals fed standard and methionine-enriched diets, as well as the changes in FA profiles during treatment with two different statins, atorvastatin or simvastatin, at therapy-equivalent doses. Moreover, the present study also examines the correlations between the FA profile and Hcy levels, which are implicated in the pathology of cardiovascular diseases.

## Materials and Methods

### Ethical Concerns

All experiment and procedures were performed in accordance with valid European guidelines regarding the experiments with animals (European Directive for welfare of laboratory animals No: 2010/63/EU). Also, our protocol of study was evaluated, checked and approved by an institutional committee (Faculty of Medical Sciences, University of Kragujevac, Serbia Ethics committee for well-being of experimental animals, Number of approval: 01-11794).

### Experimental Design

Before the experimental procedures, all rats (*n* = 72; 8 rats per group; *Wistar albino male rats*) were in adaptation period for two weeks in the animal house at the Faculty of Medical Sciences in Kragujevac. After that started dietary manipulation with three different diets separately. Animals have *ad libitum* water and food in standard laboratory controlled conditions (22 ± 2°C; light/dark cycle 12:12 h; two rats per cage). All animals were divided into the next groups:

1. Control group-Standard food for rodents (S);
2. Methionine group-Methionine-enriched food (M)
3. Methionine-enriched and folate-deficient group-Methionine enriched and deficient in deficiency in B vitamins (folic acid, B_6_ and B_12_) (MFD);
4. Atorvastatin group-Standard food for rodent plus 3 mg/kg/day i.p of atorvastatin (S+ATO);
5. Simvastatin group-Standard food for rodents plus 5 mg/kg/day i.p simvastatin (S+SIM);
6. Methionine plus atorvastatin group (M+ATO);
7. Methionine plus simvastatin group (M+SIM);
8. Methionine enriched and folate-deficient plus atorvastatin group (MFD+ATO);
9. Methionine enriched and folate-deficient plus simvastatin group (MFD+SIM);

The rats were weighed when they were assigned to treatment groups and subsequently every day. The food intake was measured daily, always at the same time during the 4-week experimental period, prior to euthanasia. The total food intake was calculated by measuring the daily consumption of food in grams as the difference between the food provided on the previous day and that left unconsumed per cage (2 rats per cage; Jeremic et al., 2018).

After 4 weeks of dietary/pharmacological treatment and short-term anesthesia by diethyl ether, blood samples were collected from the overnight (12 h)-fasted rats during while sacrificing the rats. Plasma samples were obtained from the centrifugation of heparinized blood at 1,800 g for 15 min and were stored in a −80°C freezer until further FA analysis. Additionally, serum samples were obtained by collecting blood from rats without anticoagulant followed by centrifugation.

### Diets and Drugs

In our research we used three different diets in which are different amount of methionine and B vitamins (folic acid, B_6_, and B_12_). We used standard rodent chow (3.8 g/kg of methionine, 2 mg/kg folic acid, 0.03 mg/kg B_6_, and 70 mg/kg B_12_); a diet enriched in methionine (7.7 g/kg of methionine) with no deficiency of B vitamins (2 mg/kg folic acid, 0.03 mg/ kg B_6_, and 70 mg/kg B_12_); a diet enriched in methionine and deficient in B vitamins (7.7 g/kg of methionine, 0.08 mg/kg folic acid, 0.01 mg/kg B_6_, and 0.01 mg/kg B_12_; Nikolic Turnic et al., 2018). All diets were purchased from *Mucedolla, Milan, Italy*. All drugs as analytical substances (atorvastatin and simvastatin) were obtained from Sigma Aldrich, Germany.

### Determination of Homocysteine (Hcy)

As it previously described, total serum Hcy concentrations were determined with a high-performance liquid chromatography (HPLC) procedure (Ulbink et al., 1991).

### Determination of Fatty Acid Profile in Plasma

The fatty acid profile of total lipids in plasma was determined using the described method (Glaser et al., 2010) with few modifications. Briefly, 1.5 ml HCl in methanol (3 M HCl in methanol) was added to 100 μl of plasma and heated at 85°C for 45 min. After cooling to room temperature, 1 ml hexane was added, and the sample was vortexed for 30 s and centrifuged for 10 min at 1,800x g. Then, the hexane layer, which was approximately 600 μl, was evaporated to dryness in a nitrogen stream. was dissolved in 10 μl of hexane and 1 μl was injected into a gas chromatograph (SHIMADZU 2014 168 Third Avenue Waltham, MA USA 02451). The gas chromatograph was equipped with a RESTEK Rtx 2330 capillary column (60 m × 0.25 mm × 0.2 μm). The temperature programme increased from 140 to 210°C at 3°/min. Individual fatty acids were identified compared with the retention times of PUFA-2 fatty acid methyl ester commercial standards (Supelco, Inc., Bellefonte, Pennsylvania, USA). The results are presented as the percentage of the total FA composition (Veselinovic et al., 2017).

### Estimated Elongase and Desaturase Activities (Desaturase Index)

The activities of enzymes involved in FA biosynthesis, including desaturases and elongases, were estimated as product-to-precursor ratios (desaturase and elongase index) as previously described (Tepsic et al., 2013). The estimated activity of delta-5-desaturase was calculated as the 20:4ω-6/20:3ω-6 ratio (D5-desaturase index), while the 18:3ω-6/18:2ω-6 ratio was used to estimate delta-6-desaturase activity (D6-desaturase index). Also, delta-9-desaturase, also called stearoyl-CoA desaturase (SCD) and elongase indices were obtained from 16:1/16:0 (SCD-16 index), 18:1/18:0 (SCD-18 index), and 18:0/16:0 (elongase index) ratios.

### Statistical Analyses

Statistical analysis was done by using SPSS for Windows version 22. The results are presented as the mean ± standard deviation/standard error of mean (mean ± SD/SEM). Differences between the groups were confirmed by one-way ANOVA followed by the Tukey *post hoc* test or by the Kruskal-Wallis test and Mann-Whitney *U*-test depending on the normality. Significance level was 0.05.

## Results

### Food Intake and Body Weight

We found significant differences in body weight and food intake in all groups as we previously published (Jeremic et al., 2018). We found significant differences in body weight and food intake in all groups. The MFD group consumed less food and had lower weight gain, and heart and liver weight than the S and M groups, while M had only lower weight gain and food consumption than in the S group. Additionally, a high-methionine diet caused a statistically significant increase in the heart weight/body weight and the liver weight/body weight compared to that of the control (Jeremic et al., 2018).

### Homocysteine Concentration

The highest levels of homocysteine were observed in MFD groups, and lowest levels in S groups (Table 1). Well, hyperhomocysteinemia was achieved in the groups fed the methionine-enriched special diets, and based on this observation, we confirmed normal homocysteine levels in the S, S+ATO and S+SIM groups; mild hyperhomocysteinemia (Hcy = 15–30 μmol/L) in the M, M+ATO and M+SIM groups; and severe hyperhomocysteinemia (Hcy> 31 μmol/L) in the MFD, MFD+ATO, and MFD+SIM groups (Nikolic et al., 2018; Nikolic Turnic et al., 2018) (Supplementary Table 1).

**Table 1:** Levels of homocysteine in all groups.

**Groups**	**Total homocysteine in serum (μmol/l)**		
	***S***	***M***	**MFD**
	8.11 ± 0.1	22.43 ± 0.3	63.89 ± 0.1
	S + ATO	M + ATO	MFD + ATO
	11.57 ± 3.27	16.10 ± 4.32	59.41 ± 7.12
	S + SIM	M + SIM	MFD + SIM
	11.41 ± 1.92	30.22 ± 5.71	58.51 ± 4.49

*Data are presented as mean value ± standard deviation (M ± SD) (n = 8 rats per group)*.

### Effects of Different Dietary Manipulation and Statin Treatment on Fatty Acid Composition in Total Plasma Lipids

From the plasma samples, we determined the percentage of individual FAs in the plasma lipids in all the study groups, and the results are shown in Table 2. First, we compared the FA status of rats fed different dietary regimes. When we examined the groups fed a standard diet (S, S+ATO, S+SIM), our results showed a significant increase in linoleic acid (LA, 18:2, ω-6) and eicosapentaenoic acid (EPA, 20:5, ω-3) levels in the S+ATO group compared with the controls. Additionally, we also found increased oleic acid (18:1 ω-9), LA, γ-linolenic acid (GLA, 18:3, ω-6) and EPA levels as well as decreased arachidonic (AA, 20:4, ω-6) and docosahexaenoic acid (DHA, 22:6, ω-3) levels in the simvastatin-treated rats (S+SIM) compared with the controls. The only difference between the S+ATO and S+SIM groups was lower levels of docosapentaenoic acid (DPA 22:5, ω-3) in the simvastatin-treated group. Among the groups with mild hyperhomocysteinemia (M, M+ATO and M+SIM), we observed a similar fatty acid composition in the M and M+ATO groups. However, when we compared the M+SIM and M groups, we found higher levels of stearic acid (18:0), LA and EPA and lower levels of DPA, DHA, and AA. The last two FAs were also lower in the M+SIM group compared with the M+ATO group. In the groups with severe hyperhomocysteinemia, including the MFD, MFD+ATO, and MFD+SIM groups, we found more differences between the group exposed to atorvastatin and its appropriate control group (MFD) than in the aforementioned groups. Thus, oleic acid and docosatetraenoic (DTA, 22:4, ω-6) levels were higher, while stearic acid, EPA, and DHA levels were lower than in the MFD group. Similar to the other rats that were given simvastatin, dihomo-gamma-linolenic acid (DGLA, 20:3, ω-6), EPA, DPA, and DHA levels were lower than in the MFD group. Compared with the M+ATO group, M+SIM had a lower proportion of DGLA and DPA.

**Table 2:** Plasma fatty acid profile (mol% of total fatty acids) in rats on different treatments and dietary regimes (*n* = 8 rats per group).

**Groups**	**S**	**S + ATO**	**S + SIM**	**M**	**M + ATO**	**M + SIM**	**MFD**	**MFD + ATO**	**MFD + SIM**
**Fatty acids**	**X ± SD**	**X ± SD**	**X ± SEM**	**X ± SD**	**X ± SD**	**X ± SD**	**X ± SD**	**X ± SD**	**X ± SD**
Palmitic acid	25.99 ± 0.65	23.02 ± 1.76	24.60 ± 0.79	25.51 ± 0.33	24.31 ± 0.81	25.23 ± 0.16	25.34 ± 0.43	26.16 ± 0.91	28.02 ± 0.81
Stearic acid	18.29 ± 0.56	17.90 ± 2.04	17.13 ± 0.77	17.59 ± 0.52	17.03 ± 0.49	20.53 ± 0.42^*^	17.48 ± 0.53	14.71 ± 0.55^*^	17.78 ± 0.63^#^
Palmitoleic acid	2.17 ± 0.11	1.27 ± 0.34	2.11 ± 0.23	1.73 ± 0.30	1.17 ± 0.18	2.51 ± 0.47	1.88 ± 0.17	1.50 ± 0.16	2.20 ± 0.22
Oleic acid	8.31 ± 0.57	9.12 ± 1.09	12.32 ± 0.90^***^	6.96 ± 0.36	8.18 ± 0.18	7.60 ± 0.14	7.48 ± 0.19	13.90 ± 0.22^***^	11.15 ± 0.68^**^^#^
Vaccenic acid	2.07 ± 0.15	2.36 ± 0.40	2.31 ± 0.09	1.84 ± 0.06	1.96 ± 0.08	2.13 ± 0.11	1.96 ± 0.11	2.48 ± 0.24	2.01 ± 0.12
Linoleic acid	16.43 ± 1.16	22.39 ± 4.63^*^	22.44 ± 0.47^*^	15.64 ± 2.08	17.26 ± 2.49	21.27 ± 0.23^*^	17.12 ± 1.62	17.48 ± 0.46	18.78 ± 0.99
γ-Linolenic acid	0.30 ± 0.00	0.46 ± 0.09	0.51 ± 0.04^*^	0.41 ± 0.03	0.49 ± 0.04	0.44 ± 0.04	0.32 ± 0.00	0.53 ± 0.11	0.32 ± 0.04
Dihomo-γ-linolenic acid	0.95 ± 0.11	1.30 ± 0.30	1.09 ± 0.14	1.03 ± 0.12	0.94 ± 0.02	1.00 ± 0.13	1.12 ± 0.11	1.35 ± 0.10	0.64 ± 0.09^*^^##^
Arachidonic acid	20.61 ± 1.53	17.24 ± 3.80	14.12 ± 1.21^*^	23.71 ± 1.89	22.49 ± 1.84	15.17 ± 0.41^**^^#^	21.42 ± 2.09	17.53 ± 0.68	16.38 ± 1.65^*^
Docosatetraenoic acid	0.40 ± 0.02	0.37 ± 0.02	0.35 ± 0.02	0.46 ± 0.02	0.54 ± 0.04	0.51 ± 0.07	0.43 ± 0.03	0.76 ± 0.11^*^	0.43 ± 0.08
α-Linolenic acid	0.30 ± 0.03	0.41 ± 0.13	0.32 ± 0.03	0.65 ± 0.03^aa^	0.51 ± 0.04	0.60 ± 0.04	0.45 ± 0.14	0.47 ± 0.04	0.33 ± 0.04
Eicosapentaenoic acid	0.39 ± 0.05	0.75 ± 0.23^*^	0.52 ± 0.06^*^	0.27 ± 0.03	0.28 ± 0.08	0.49 ± 0.06^*^	0.51 ± 0.11	0.09 ± 0.06^*^^*^	0.29 ± 0.04^*^
Docosapentaenoic acid	0.49 ± 0.04	0.76 ± 0.12	0.40 ± 0.05^##^	0.83 ± 0.06^aa^	0.84 ± 0.03	0.61 ± 0.03^*^	0.62 ± 0.01	0.72 ± 0.06	0.39 ± 0.02^*^^###^
Docosahexaenoic acid	3.32 ± 0.50	2.68 ± 0.51	1.78 ± 0.16^***^	3.38 ± 0.17	4.00 ± 0.32	1.77 ± 0.08^***^^###^	3.87 ± 0.04	2.26 ± 0.14^***^	1.30 ± 0.14^***^^#^

*Date are expressed as the mean ±SD*.

* *p < 0.05*,

** *p < 0.01*,

*** *p < 0.001 compared to appropriate control group in the same dietary regime*;

# *p < 0.05*,

## *p < 0.01*,

### *p < 0.001 compared to atorvastatin group in the same dietary regime*.

aa *p < 0.01 compared with S group*.

As is shown in Table 3, there were no significant differences in the groups regarding their estimated D6-desaturase and elongase activities. Elevated SCD-18 index, as an estimated activity of this enzyme, was detected in the S+SIM group compared with its control group S and in the MFD+ATO group compared with both the MFD and MFD+SIM groups. Furthermore, simvastatin elevated indices of SCD-16 in all groups, while atorvastatin reduced these indices, although these changes were not statistically significant in the MFD groups. Both statins decreased D5-desaturase index in the animals fed a standard diet, as well as in the M+SIM group, but increased the estimated activity of this enzyme in the MFD+SIM group.

**Table 3:** The estimated plasma desaturases and elongase activities in rats on different treatments and dietary regimes (*n* = 8 rats per group).

**Groups**	**S**	**S + ATO**	**S + SIM**	**M**	**M + ATO**	**M + SIM**	**MFD**	**MFD + ATO**	**MFD + SIM**
**Enzyme index**	**X ± SD**	**X ± SD**	**X ± SEM**	**X ± SD**	**X ± SD**	**X ± SD**	**X ± SD**	**X ± SD**	**X ± SD**
SCD-16	0.08 ± 0.01	0.06 ± 0.01^*^	0.09 ± 0.02^#^	0.07 ± 0.01	0.05 ± 0.00^*^	0.10 ± 0.02^*^^#^	0.07 ± 0.02	0.06 ± 0.01	0.08 ± 0.01
SCD-18	0.46 ± 0.02	0.52 ± 0.12	0.74 ± 0.09^*^	0.40 ± 0.03	0.48 ± 0.01	0.47 ± 0.01	0.43 ± 0.02	0.95 ± 0.03^***^	0.64 ± 0.06^##^
D6-desaturase	0.02 ± 0.00	0.02 ± 0.00	0.02 ± 0.00	0.03 ± 0.00	0.03 ± 0.01	0.02 ± 0.00	0.02 ± 0.00	0.03 ± 0.01	0.02 ± 0.00
D5-desaturase	22.22 ± 1.85	13.31 ± 0.08^**^	13.91 ± 1.84^**^	24.23 ± 3.80	23.92 ± 1.52	16.80 ± 2.33^#^	19.91 ± 3.62	13.18 ± 0.94	23.44 ± 3.39^*^^#^
Elongase	0.70 ± 0.03	0.79 ± 0.15	0.70 ± 0.05	0.69 ± 0.01	0.70 ± 0.04	0.82 ± 0.02	0.69 ± 0.03	0.56 ± 0.03	0.64 ± 0.04

*Date are expressed as the mean ± SD. SCD, Stearoyl-CoA desaturase; D5-desaturase, delta-5 desaturase; D6-desaturase, delta-6 desaturase*.

* *p < 0.05*,

** *p < 0.01*,

*** *p < 0.001 compared to appropriate control group in the same dietary intervention*;

# *p < 0.05*,

## *p < 0.01*,

### *p < 0.001 compared to atorvastatin group in the same dietary intervention*;

aa *p < 0.01 compared with S group*.

### Effects of Different Dietary Manipulation and Statin Treatments on Total SFA, MUFA, and PUFA

**Figure 1:**
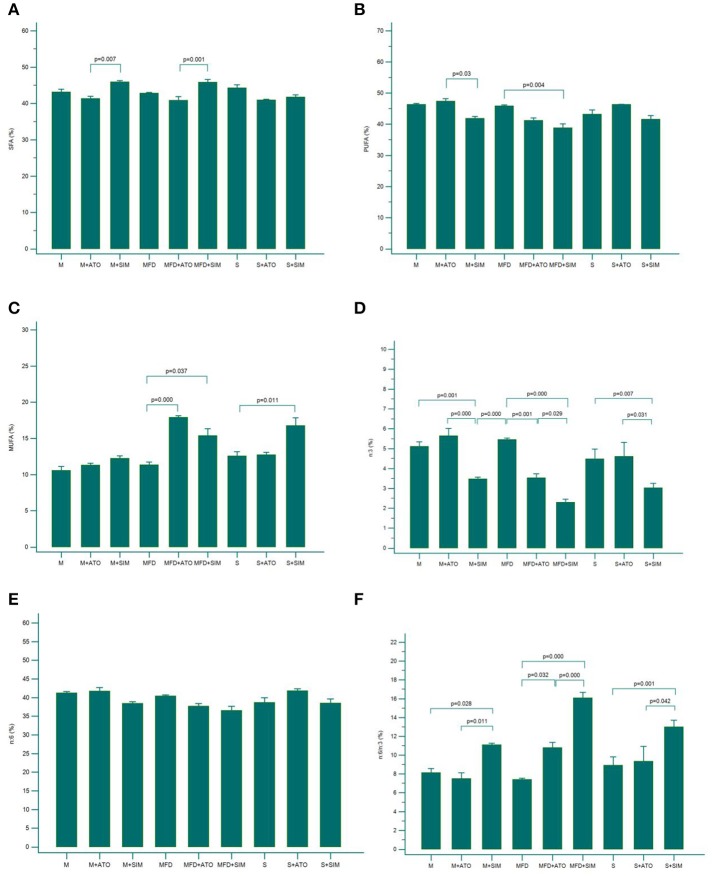
Levels of SFA **(A)**, PUFA **(B)** and MUFA **(C)**, ω−3 PUFA **(D)**, ω−6 PUFA **(E)**, and ω−6 /ω-3 ratio **(F)** in all investigated groups (*n* = 8 rats per group). Results are expressed as mean ± SEM. SFA, saturated fatty acids; MUFA, monounsaturated fatty acids; PUFA, polyunsaturated fatty acids.

Animals with Hhcy (the M and MFD groups, Figure 1A) treated with simvastatin had significantly higher SFA levels than the groups taking atorvastatin. Simvastatin also increased MUFA levels in the S group. Both statins elevated MUFA levels in the MFD group (Figure 1C), while PUFA levels were lower in the MFD+SIM group than in its control (MFD), as well as in the M+SIM group compared with the M+ATO group (Figure 1B).

### Effects of Different Dietary Manipulation and Statin Treatments on Total ω-3 and ω-6 Amounts and the ω-6/ ω-3 Ratio

The levels of total ω-3 PUFAs in plasma samples were significantly decreased in all the groups treated with simvastatin (S+SIM, M+SIM and MFD+SIM) compared to the appropriate control groups and groups treated with atorvastatin (Figure 1D). Accordingly, the ω-6/ω-3 ratios were significantly higher in all the groups on simvastatin compared with their controls and the atorvastatin groups (Figure 1F). Additionally, in the groups with severe hyperhomocysteinemia (MFD group), atorvastatin significantly reduced ω-3 levels and increased the ω-6/ω-3 ratio (Figures 1D–F).

### Correlation Analysis of Hcy Levels and the Fatty Acid Profile

**Figure 2:**
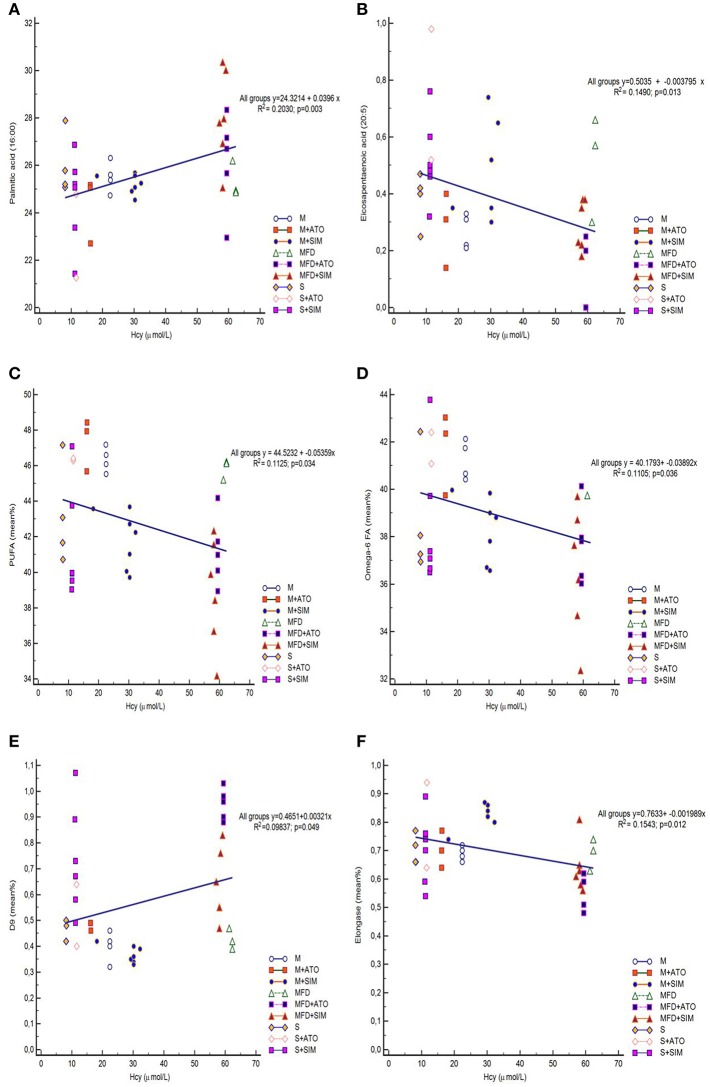
Regression analyses. Confirmed correlation was observed between Hcy and: Palmitic acid **(A)**, EPA **(B)**, PUFA **(C)**, ω−6 PUFA **(D)**, SCD-18 index **(E)**, and Elongase **(F)** among all groups (8 rats per group). EPA, eicosapentaenoic acid; PUFA, polyunsaturated fatty acids; SCD-18, Stearoyl-CoA desaturase (18:1/18:0).

Figure 2 displays only variables that were significantly correlated with Hcy levels. Only palmitic acid and SCD-18 index were moderately positively correlated with homocysteine levels in all the groups (Figures 2A,E). In contrast, EPA, total PUFA, and ω-6 FA levels and elongase index were weakly negatively correlated with homocysteine levels (Figures 2B–D,F).

## Discussion

The present study aimed to examine the associations of statins, the FA profile, and homocysteine levels, which are implicated in the pathology of cardiovascular diseases.

Our experimental model of diet-induced Hhcy is an absolutely adequate model for Hhcy, as most chronic changes associated with homocysteine levels are related to diet in humans.

The two different dietary regimes induced different degrees of Hhcy: moderate and severe. In all groups, statins did not change the levels of homocysteine, which is similar to our previously published results; thus, we could clearly comment on the effects of these lipid-lowering drugs on the FA profile (Nikolic et al., 2018; Nikolic Turnic et al., 2018). The only difference in the FA profile depending on the dietary treatment was observed in the level of ALA, which was slightly higher in the group of animals fed a diet enriched in methionine with no deficiency in B vitamins (M). This confirms that all the alterations in FA composition of plasma lipids are related to statin treatments.

There is a large pool of data supporting the pleiotropic effects of statins on the cardiovascular system (Colhoun et al., 2004; Fauchier et al., 2008; Nassief and Marsh, 2008). Many researchers have reported improved heart function, decreased cholesterol levels and reduced CVD symptoms (Baszczuk et al., 2014; Zhang et al., 2014). Other studies suggest benefits of statins in the reduction of stroke risk, atrial fibrillation, and incidence of myocardial infarction, as well as for the treatment of chronic kidney disease, rheumatologic diseases and multiple sclerosis (Colhoun et al., 2004; Schaffer et al., 2014). Nevertheless, there are no data focused on the specific roles of the FA contents in all these conditions during statin treatment. Studies evaluating the effects of statins on plasma FA metabolism in humans are sparse and published data are contradictory (Risé et al., 2001; Bird et al., 2018).

In the first part of this study, we examined the effects of different dietary regimes and atorvastatin and simvastatin on individual fatty acids. Total ω-3 PUFA levels significantly decreased and the ω-6/ω-3 ratio in plasma samples increased in the simvastatin-treated groups compared with both the control and atorvastatin-treated groups. Furthermore, in the MFD group with severe Hhcy, atorvastatin treatment lowered ω-3 levels, which induced a higher ω-6/ω-3 ratio. Simultaneously, statins did not change ω-6 PUFA levels. However, level of LA was mostly higher and AA was lower in statin treated groups, suggesting decreased conversion from LA to AA. Considering that AA is the most pro-inflammatory FA and a precursor of several inflammatory eicosanoids, these findings suggest that statins, especially simvastatin, may have anti-inflammatory effects. The other possibility is that AA is rapidly converted into eicosanoids, thereby promoting inflammation. The clarification of the effects of statins on inflammation require further investigation, but statin use obviously increase the ω-6/ω-3 ratio (Takahashi et al., 2017), which usually result in a net increase in inflammation and thrombogenesis (de Lorgeril et al., 2013), as well as further CVD development and progression. Taking into account the importance of high ω-3 PUFA levels not only for cardiovascular health, but also for other chronic diseases, significant decrease in ω-3 PUFA levels in statin use found in this study, is of a particular importance.

It is interesting that elevated SCD-18 index, as an estimation of desaturase activity, was found in the simvastatin groups fed a standard diet and a diet enriched with methionine and deficient in B vitamins (MFD), but in severe Hhcy atorvastatin treatment increased SCD-18 index even more than simvastatin. Simvastatin also increased SCD-16 index in the M group, while atorvastatin reduced the estimated activity of this enzyme S+ATO and M+ATO groups. Both statins decreased D5-desaturase index in the animals fed a standard diet. Simvastatin treatments decreased D5-desaturase index in the control group and in mild Hhcy but increased the activity of this enzyme in severe hyperhomocysteinemia. In human studies, simvastatin increased D5-desaturase activity in dyslipidaemia (Jula et al., 2005; Nozue and Michishita, 2015), but atorvastatin decreased D5-desaturase activity in metabolic syndrome model rats (Al Mamun et al., 2017). It is important to point out that there were no differences among the groups in the estimated activities of D6-desaturase and elongase. This can lead to changes in the relative proportions of longer chain PUFAs.

Meta-analysis of randomized control trials indicated that high consumption of ω-3 PUFA decreases plasma Hcy concentrations (Huang et al., 2011). Moreover, the same authors showed that ω-3 PUFA upregulates mRNA expression, while n-6 PUFA downregulates the mRNA expression of key genes involved in homocysteine metabolism (Huang et al., 2013). These mechanisms could be one of the possible reasons for the altered FA levels during severe Hhcy. Other authors (Severus et al., 2001) examined the significant roles of Hcy in homocysteine-related cardiovascular disease treated with ω-3 PUFA and concluded that homocysteine and FAs are two very important factors that may have a causal relationship.

Previous data confirmed that Hcy as a thiol could modulate cardiovascular function and act through various mechanisms, such as induce endothelial dysfunction leading to the alterations in arterial structure. Some of the proposed mechanisms include a proliferation of vascular smooth muscle cells, endothelial dysfunction, oxidative damage, collagen synthesis, and the deterioration of the arterial wall elastic material (Durand et al., 2001; Piolot et al., 2003; Schaffer et al., 2014).

In addition to ω-3 and ω-6 PUFAs, other fatty acids in our study were also changed. Simvastatin treatments induced an increase in SFAs in animals with Hhcy compared to atorvastatin. Both statins elevated MUFAs in the group with severe Hhcy. In the second part of this study, we correlated the levels of Hcy and FAs in blood samples. Levels of EPA, total PUFAs and ω-6 PUFA negatively correlated, while saturated palmitic acid and SCD-18 moderately positively correlated with homocysteine levels. According to our results, homocysteine is a biomarker of cardiovascular disorder, and high levels of ω-3 is an indicator of cardiovascular health. We can indirectly observe that folate, B_12_ and B_6_ intake were associated with both serum ω-3 and ω-6 PUFAs and homocysteine levels. In line with this result, an observational study suggested that higher dietary intake of ω-3 PUFAs was associated with decreased plasma homocysteine levels (Berstad et al., 2007). Regarding the levels of ω-6 FAs, some studies have examined the associations between plasma ω-6 PUFA levels and Hcy concentrations (Huang et al., 2012a,b). Some authors have concluded that plasma ω-6 PUFA was significantly positively associated with plasma homocysteine in some conditions, such as in vegetarians, diabetes patients, and the general population (Huang et al., 2012a,b). In the other studies, however, ω-6 PUFA was not associated with plasma homocysteine in omnivores, while we found a slight negative correlation. The results from this study are in accordance with the assumptions that omega-6 FAs have negative effects on the cardiovascular system as well as homocysteine; thus, the positive connection between Hcy and omega-6 FAs in our study is completely expected. Additionally, simvastatin exerts stronger effects on FA status during Hhcy than atorvastatin.

## Conclusion

Statins considerably affect FA status in animals with hyperhomocysteinemia, in particular simvastatin. They decrease the levels of cardioprotective ω-3 PUFA. Lower serum Hcy concentrations were observed in association with higher EPA, ω-6 and total PUFA levels. Moreover, higher levels of Hcy correlate with elevated atherogenic palmitic acid.

These findings pointed for the significant role of ω-3 FAs in lowering homocysteine concentrations, as well as the role of statins in FA status and changing the risk for CVD in general as a consequence. Our results also suggest that statin use should be followed by ω-3 PUFA supplementation, which is obviously needed during the statin treatments.

Prospective studies are required to clarify whether a higher ω-3 or ω-6 FAs status at baseline is associated with the future decrease in blood homocysteine levels and the risk of homocysteine-related diseases.

## Data Availability

All the datasets for this study are available on request to the corresponding author.

## Ethics Statement

This study was carried out in accordance with the recommendations of European Directive for welfare of laboratory animals No: 2010/63/EU and Good Laboratory Practice (GLP) principles. The protocol for the current study was approved by the Ethics committee for experimental animal well-being of the Faculty of Medical Sciences at the University of Kragujevac, Serbia (No: 01-11794).

## Author Contributions

TN, JJ, IM, and StB performed the experiments and collected data. VZ, IS, TR, and SeB performed statistical analyses. AA, VV, SP, and DR-M performed biochemical analyses and collected biochemical data from study. DD and VJ designed the study and performed experiments. All authors contributed in interpretation of results and to writing the manuscript.

### Conflict of Interest Statement

The authors declare that the research was conducted in the absence of any commercial or financial relationships that could be construed as a potential conflict of interest.
